# Acquired Hypogonadotropic Hypogonadism (AHH) in Thalassaemia Major Patients: An Underdiagnosed Condition?

**DOI:** 10.4084/MJHID.2016.001

**Published:** 2016-01-01

**Authors:** Vincenzo De Sanctis, Heba Elsedfy, Ashraf T Soliman, Ihab Zaki Elhakim, Alessia Pepe, Christos Kattamis, Nada A. Soliman, Rania Elalaily, Mohamed El Kholy, Mohamed Yassin

**Affiliations:** 1 Pediatric and Adolescent Outpatient Clinic, Quisisana Hospital, Ferrara, Italy; 2 Department of Pediatrics, Ain Shams University, Cairo, Egypt; 3 Department of Pediatrics, Division of Endocrinology, Alexandria University Children’s Hospital, Alexandria; 4 Cardiovascular MR Unit, Fondazione G. Monasterio CNR-Regione Toscana and Institute of Clinical Physiology, Pisa, Italy; 5 First Department of Paediatrics, University of Athens, Athens, Greece; 6 Ministry of Health, Alexandria, Egypt; 7 Department of Primary Health Care, Abu Nakhla Hospital, Doha, Qatar; 8 National Center for Cancer Care and Research, Department of Hematology and BMT, Hamad Medical Corporation, Doha, Qatar

## Abstract

**Introduction:**

In males, acquired hypogonadotropic hypogonadism (AHH) includes all disorders that damage or alter the function of gonadotropin-releasing hormone (GnRH) neurons and/or pituitary gonadotroph cells. The clinical characteristics of AHH are androgen deficiency and lack, delay or halt of pubertal sexual maturation. AHH lead to decreased libido, impaired erectile function, and strength, a worsened sense of well-being and degraded quality of life (QOL).

**Patients and methods:**

We studied 11 adult men with thalassemia major (TM) aged between 26 to 54 years (mean ± SD: 34.3 ± 8.8 years) with AHH. Twelve age- and sex-matched TM patients with normal pubertal development were used as a control group. All patients were on regular transfusions and iron chelation therapy. Fasting venous blood samples were collected two weeks after transfusion to measure serum concentrations of IGF-1, free thyroxine (FT4), thyrotropin (TSH), cortisol, luteinizing hormone (LH), follicle stimulating hormone (FSH), total testosterone (TT), prolactin and estradiol (E2), glucose, urea, creatinine and electrolytes (including calcium and phosphate). Liver functions and screening for hepatitis C virus seropositivity (HCVab and HCV-RNA) were performed. Iron status was assessed by measuring serum ferritin levels, and evaluation of iron concentrations in the liver (LIC) and heart using MRI- T2*. Bone mineral density was measured at the lumbar spine (L1–L4) for all patients with AHH by dual energy X-ray absorptiometry (DXA) using Hologic QDR 4000 machine.

**Results:**

The mean basal serum LH and FSH concentrations in AHH patients were 2.4 ± 2.2 IU/L and 1.2 ± 0.9 IU/L respectively; these, values were significantly lower compared to the control group. Semen analysis in 5 patients with AHH showed azoospermia in 3 and oligoasthenozoospermia in 2. The percentage of patients with serum ferritin level >2000 ng/ml (severe iron load) was significantly higher in AHH patients compared to controls, 5/11 (45.4 %) versus1/12 (8.3%), p=0.043. Heart iron concentrations (T2* values) were significantly lower in AHH patients compared to controls (p=0.004). Magnetic resonance imaging in the 3 azoospermic patients revealed volume loss and reduction of pituitary signal intensity. Heart T2* values were significantly reduced in the AHH group vs. the controls (p=0.004). On the other hand, liver iron concentration (mg/g dry weight) was not different between the two groups of TM patients. Using DXA, 63.6 % (7/11) of patients with AHH were osteoporotic, and 36.3 % (4/11) were osteopenic.

**Conclusions:**

In this cohort of thalassemic patients iron overload and chronic liver disease appear to play a role in the development of AHH. Treatment of AHH in TM patients is a vital and dynamic field for improving their health and QOL. Early identification and management of AHH are very crucial to avoid long-term morbidity, including sexual dysfunction and infertility. Therapy aims to restore serum testosterone levels to the mid–normal range. Many exciting opportunities remain for further research and therapeutic development.

## Introduction

In males, acquired hypogonadotropic hypogonadism (AHH) includes all postnatal disorders that damage or alter the function of gonadotropin-releasing hormone (GnRH) neurons and/or pituitary gonadotroph cells. The anatomical, histological and functional changes of AHH encompass a vast range of causes including infiltrative and infectious pituitary lesions (e.g., hemosiderosis, sarcoidosis and histiocytosis), hyperprolactinemia, brain injury and pituitary irradiation.^1^–^4^

In adolescents, the clinical presentations of AHH include lack, delay and/or halt of pubertal sexual maturation. Adults with AHH have decreased libido, erectile dysfunction, worsened sense of well-being and lower quality of life (QOL). Physical examination is usually normal in hypogonadism of recent-onset. In long-standing AHH, diminished facial and body hair decreased muscle mass, and appearance of fine facial wrinkles, gynecomastia, and small testes are observed. Spermatogenesis is impaired, and the volume of ejaculate is decreased only when gonadotropins and testosterone levels are very low. Low serum concentrations of testosterone and gonadotropins confirm the diagnosis of AHH.^1^–^7^

Adult male patients with TM, on frequent blood transfusions, are predisposed to develop AHH. The prevalence of AHH in TM depends mainly on the age composition of the thalassemia cohort and the degree of compliance with blood transfusion and mainly chelation programs.

We report 11 adult men with thalassemia major (TM) and AHH. They presented with loss of libido and/or infertility; reduced shaving frequency; erectile or ejaculatory dysfunction; diagnosis of AHH was confirmed by appropriate laboratory tests.^8^,^9^ They were selected from a cohort of adult TM patients with significant compliance to blood transfusion and efficient implementation of chelation programs.

## Patients and Methods

### Setting and study design

The study started at the beginning of 2009 by VDS, (Coordinator of International Network of Clinicians for Endocrinopathies in Thalassemia and Adolescent Medicine ICET-A) at the Thalassaemia Centre of Ferrara^10^ and was completed by the end of August 2015 at the Quisisana Pediatric and Adolescent Outpatient Clinic of Ferrara. During this period, 11 adults with TM (aged between 26 to 54 years, mean ± SD: 34.3 ± 8.8 years) were diagnosed with hypogonadotropic hypogonadism and were studied.

All patients had spontaneous, full pubertal development. At presentation, patients complained of sexual dysfunction and/or fertility problems, loss of libido or infertility, and erectile or ejaculatory dysfunction (diminished erectile quality and frequency; diminished early morning erections; decreased or watery semen production). There was no history of anosmia, cryptorchidism, brain injury, exposure to chemicals or drug abuse. Patients had no family history of infertility, mental, physical or pubertal development retardation.

A randomly selected 12 age- and sex-matched TM patients with spontaneous and full pubertal development, who visited our outpatient clinics for clinical and laboratory endocrine assessment served as controls.

Ethical approval for the study was obtained in accordance with local institutional requirements in accordance with the Declaration of Helsinki (http://www.wma.net). All procedures were carried out with the adequate understanding and consent of patients.

### Exclusion criteria

Exclusion criteria included: 1) treatment with androgens or anabolic steroids within the last 3 months; 2) hyperprolactinemia or treatment with prolactin-lowering drugs; 3) mental illness (depression, anxiety disorders, eating disorders and addictive behaviors). 4) acute diseases; 5) accidental severe head trauma and brain injury; 6) alterations in nutritional status with significant loss of weight and/or the presence of depression; 7) smoking of more than 15 cigarettes/day or alcohol abuse (more than three glasses of wine/day); and 8) increased estrogen levels secondary to liver disease.

### Research design

Extensive history was taken including data on associated complications of thalassemia and current medications. Thorough physical examination was completed including anthropometry (weight, height, BMI), vital signs (blood pressure, heart rate) and genital status. Body mass index (BMI) was calculated (weight in Kg/height in m^2^). A subject was considered overweight when the BMI was between 25 and 30 and obese above 30.^11^ Age at first transfusion, interval between transfusions, type, and compliance to iron chelation and associated endocrine complications were recorded.^11^

### Blood sampling and analytical procedures

All blood samples were collected in the morning (08.00 – 09.00 am), after an overnight fast, and 2 weeks after blood transfusion. The circulating concentrations of IGF-1, free thyroxine (FT4), thyrotropin (TSH), cortisol, luteinizing hormone (LH), follicle stimulating hormone (FSH), total testosterone (TT), prolactin, estradiol (E2), glucose, urea, creatinine and electrolytes (including calcium and phosphate) were measured.

To exclude severe liver injury and dysfunction, serum concentrations of alanine aminotransferase (ALT), gamma glutamyl transferase (γ GT), alkaline phosphatase (ALP), total and direct bilirubin, total proteins, albumin, prothrombin time (PT) and international normalization ratio (INR) were measured. Screening assays for hepatitis C virus seropositivity (HCVab and HCV-RNA) were performed applying routine laboratory methods.

Plasma total IGF-1 was measured by a chemiluminescent immunometric assay (CLIA) method (Nichols Institute Diagnostics, San Juan, CA). The assay was performed after separation of IGF-1 from binding proteins by Liaison® autoanalyzer (DiaSorin SpA, Saluggia, Italy). A detailed description of the method and the evaluation of the results have been recently published.^12^ The sensitivity of the test was 6 ng/ml, whereas the intra- and interassay coefficients of variation (CVs) of our in-house pooled serum control sample were 4.8% and 6.7 %, respectively. The reported analytical sensitivity of this assay was from 6 to 25 ng/ml (normal values set at the 2.5th–97.5th percentile were: 95.6–366.7 ng/ml for ages 25 to 39 yrs, 60.8–297.7 ng/ml for 40 to 59 yrs).^12^

The other hormonal, biochemical and hematogical parameters were determined using commercially available automated chemiluminescence immunoassay and other systems. The intra- and interassay CV for all methods were < 5.8% and < 7.8%, respectively.

### Definitions of endocrine disorders

Low blood testosterone levels and low pituitary gonadotropin levels (LH and FSH) indicated a diagnosis of HH. (7,9,11) Diabetes and secondary TSH deficiency were defined as previously described.^11^ Adrenal insufficiency was diagnosed if basal cortisol was 3.5 μg/dl (98 nmol/liter) or less.^13^ Hyperprolactinemia was defined as a basal level greater than the locally derived normal assay reference range.

### Assessment of iron overload

Iron overload was assessed by direct and indirect methods. At the beginning of the study, it was evaluated by measuring serum ferritin level. Iron status was classified as mild (ferritin < 1000 ng/ml), moderate (ferritin >1000 ng/ml and < 2000 ng/ml) or severe (ferritin >2000 ng/ml).^14^ Commercial reagents were used for the determination of serum ferritin levels based on an immune- enzymatic method.

In 6 TM patients with AHH and 8 patients without AHH, heart iron was assessed by Magnetic resonance imaging (MRI) using a 1.5 T scanner (GE Signa/Excite HD, Milwaukee, WI, USA) within the Myocardial Iron Overload in Thalassemia (MIOT) network, where MRI scans are performed using homogeneous, standardized and validated procedures.^14^,^15^ A traditional cut-off value of heart T2* > 20 ms was considered normal.^15^ In the same patients, liver iron concentration (LIC) was assayed by MRI.^16^ Liver T2* values were converted into MRI liver iron content (LIC) values sing the calibration curve introduced by Wood et al.^18^ In two TM patients with AHH and one patient without AHH, LIC was quantified by biopsy using atomic absorption spectrophotometry or Superconducting Quantum Interference Device (SQUID) and the LIC values were expressed as mg/g dry weight (dw). (18) LIC (mg Fe/gr dw) was classified as mild (LIC > 3 and < 7), moderate (LIC > 7 and < 14) and severe (LIC > 14).^14^

Bone mineral density (BMD) at the lumbar spine (L1–L4) was assessed for all AHH patients using dual energy x-ray absorptiometry (DXA) by Hologic QDR 4000 machine (Bedford, MA).

Osteopenia or osteoporosis was defined according to World Health Organization (WHO) criteria, based on BMD expressed as Z-score: osteopenia (Z-score between −1 to −2.5 SD) and osteoporosis (Z-score < −2.5 SD).^19^ Z-score is the number of standard deviations above or below what is normally expected for someone with the same age, sex, and ethnic origin. Osteoporosis is a common disorder of reduced bone strength that predisposes to an increased risk for fractures in older individuals.

Three TM patients with AHH underwent pituitary MRI using a 1.5T scanner (Sonata, Siemens Medical, Erlanger, Germany). Pituitary-to-fat signal intensity ratios (SIR) were calculated from coronal T2-weighted images. Estimated pituitary volumes were measured using pituitary height, width, and length on T1-weighted images.

### Statistical analysis

Standard computer program SPSS for Windows, release 13.0 (SPSS Inc, Tulsa, IL, USA) was used for data entry and analysis. All numeric variables were expressed as mean ± standard deviation (SD). Comparison of different variables in the two groups was done using unpaired - student t-test and Mann-Whitney test for normal and nonparametric variables respectively. Chi-square (χ^2^) test was used to compare the frequency of qualitative variables among the different groups. Pearson’s and Spearman’s correlation tests were used to study correlations between variables with parametric and non-parametric distributions respectively. p < 0.05 was considered significant.

## Results

### Patients’ characteristics

All patients were on regular transfusions (pre-transfusional hemoglobin level 9 ± 0.3 g/dl) but different iron chelation regimes therapy. Ten patients were on deferoxamine (DFO) 30–45 mg/kg body weight, 4–6 days a week by slow subcutaneous infusion by the pump. Nine patients were on oral deferiprone (DFP) 75 mg/kg body weight daily. Two patients were on combined deferiprone plus deferoxamine 75 mg/kg body weight daily and 40 mg/kg body weight, 3 days a week, by slow subcutaneous infusion and 2 on oral deferasirox (DFX) 25–30 mg/kg body weight daily. Chelation therapy has been changed over time. Treatment with intramuscular DFO was available since 1969, DFP since 1995 and DFX since 2007.

The baseline demographic and clinical data of the AHH and non-AHH groups of adult patients with TM are summarized in Table 1. The mean (± SD) age and BMI did not differ between TM patients with and controls (without) AHH. All AHH patients had spontaneous, and full pubertal development, and 2 reported previous paternity.

The presenting symptoms in patients with AHH included: loss of libido (7 patients), infertility (2 married patients); diminished erectile quality and frequency, diminished early morning erections (9 patients); fatigue (1 patient); ejaculatory dysfunction, decreased or watery semen production (11 patients). Symptoms were present for 2 – 9 months before endocrine evaluation; and were attributed to the “iron overload, chronic diseases itself, low hemoglobin level, liver dysfunction, associated endocrine complications (diabetes, hypothyroidism)”. Patients had no history of anosmia, cryptorchidism, head injury, exposure to chemicals, or alcohol and drug abuse. They had no family history of infertility, or mental, physical or pubertal development retardation.

The demographic and chelation data, the co-existent endocrinopathies and the biochemical, hormonal and iron-load results of patients with AHH and controls are summarized in tables 1 and 2.

TM patients with AHH were taller compared to non-AHH patients (169.5 ± 5.8 cm vs. 160.5 ± 8.7 cm; p: 0.008) (Table 1). In all patients, genital examination revealed a normal adult-sized penis and testis volume between 12–25 mL. All patients with AHH had undergone splenectomy due to hypersplenism and/or massive splenomegaly, versus 50% of the control group.

The mean baseline serum luteinizing hormone and follicle-stimulating hormone concentrations in AHH patients were 2.4 ± 2.2 IU/L (95% confidence interval for mean: 0.9 –3.8 IU/L) and 1.2 ± 0.9 IU/L (95% confidence interval for mean: 0.6–1.7 IU/L), respectively. These values were significantly lower compared to controls. The circulating concentrations of TT and IGF-I were significantly lower in patients with AHH group versus controls. In all patients with AHH plasma IGF- 1 concentrations were below the 2.5^th^ percentile of normal Italian population of the same age range. Growth hormone after stimulation test was not assessed. In patients with AHH serum estradiol and prolactin concentrations were normal. 58.3% of patients with AHH and 36.3% of controls had other associated endocrinopathies. (Table 1). None had a basal cortisol equal or below 3.5 μg/dl (98 nmol/liter).

Five patients with AHH provided semen specimens for testing. Three had azoospermia, and two had severe oligoasthenozoospermia. Magnetic resonance imaging in three azoospermic patients revealed pituitary volume loss and a reduction in signal intensity.

Osteoporosis was diagnosed in 63.6 % (7/11) and osteopenia in 36.3 % (4/11) of the TM patients with AHH using DXA scan (Table 1).

Patients with AHH had significantly higher serum γGT concentrations and non-significantly higher ALT concentrations compared to controls. HCV-RNA seropositive were 8/11(72%) of patients with AHH versus 4/9 (44%) of controls (p=0.059) (Table 1). Serum ferritin, albumin, and alkaline phosphatase concentrations did not differ between the two groups (Table 2).

### Assessment of iron overload

Serum ferritin level >2000 ng/ml (severe iron overload) was present in 5 patients (45.4 %) with AHH versus 1 patient (8.3%) without AHH (Table 2). Heart T2* values were significantly reduced in the AHH group vs. controls (p=0.004). On the other hand, liver iron concentration (mg/g dry weight) was not different between the two groups.

### Other correlations

A significant correlation was found between FSH and total testosterone (r = 0.632, p=0.002); total testosterone and IGF1 (r = 0.590, p=0.005) and IGF-1 and ϒGT (r = − 0.422, p=0.050). Total testosterone was correlated significantly with the height (r = − 0.552, p=0.008). No correlation was observed between serum ferritin on the one hand and serum LH, FSH, and TT concentrations on the other hand.

## Discussion

Hypogonadotropic hypogonadism (HH) is the most frequent endocrinopathy in transfused patients with TM.^11^,^20^ Patients with TM usually suffer from iron overload as a consequence of frequent transfusions and ineffective erythropoiesis. Iron has a catalytic role that produces powerful reactive oxidant species (ROS) and free radicals, which leads to oxidative damage.^21^ The sensitivity of different organs to accumulate iron varies considerably. Iron accumulates in tissues with high levels of transferrin receptor such as liver, heart and endocrine glands. The anterior pituitary gland is particularly sensitive to free radical oxidative stress that may impair gonadotropins and growth hormone (GH) secretion. Consequently thalassemic patients with marked hemosiderosis are predisposed to develop hypogonadism and short stature.^20^

The best predictor of pituitary iron overload is the detection of decreased signal intensity of the anterior lobe of the pituitary gland on T2-weighted MRI.^22^ Unfortunately at the time of the study this technique was available only for the three azoospermic TM patients with AHH.

Our patients with AHH had significantly elevated levels of γ GT and higher prevalence of HCV-RNA seropositivity and associated endocrinopathies compared to controls without AHH. These findings point to the importance of liver disease and associated endocrine disorders as important contributing factors in the etiology of HH.^11^,^20^

The study also strongly supports that AHH in TM patients is associated with a reduced heart T2* (ms). Myocardial T2* values < 20ms (1.1mg/g.dw) indicate cardiac iron overload. The vast majority of patients who present with heart failure caused by cardiac iron overload have T2* < 10 ms and low T2* values are powerful predictors of the subsequent development of cardiac failure. Therefore early detection of low myocardial T2* (< 20 ms) is important for an early treatment of cardiac iron overload and prevention of cardiac failure.^16^,^17^,^22^

Recently, it was shown that blood transfusion produces significant acute changes in the hormonal milieu and sperm parameters of patients with iron overload.^23^,^24^ Moderate hypoxia has been shown to decrease gonadotropin secretion within 2 days of arrival at moderate altitude.^25^

Our TM patients with AHH had significantly lower serum LH, FSH, testosterone concentrations and higher γ GT levels compared with control patients without AHH. Moreover, they showed significantly higher levels of hepatic iron overload and higher prevalence of serum ferritin level > 2000 ng/ml. This abnormal iron overload status similarly involves the pituitary gland and causes progressive, irreversible damage to LH, FSH, and GH secretion. However, a potential negative effect of chronic intermittent anemia on gonadotropins secretion cannot be excluded, even though our patients were on regular transfusion regimens (pre-transfusional Hb level 9.0 ± 0.3 g/dl). A pre-transfusion Hb level of 9 g/dl may not be capable of suppressing adequately bone marrow (BM) activity and intestinal absorption of iron. In support of this view, all patients with AHH had undergone splenectomy due to hypersplenism and/or massive splenomegaly, versus 50% of the control group. This difference implied that transfusion therapy was less efficient in suppressing BM hyperactivity and extramedullary hemopoiesis that leads to marked splenic enlargement.

An accurate assessment of the prevalence rate of AHH in adults with TM is difficult, and under-diagnosis is common. There are few data in the literature studying this new emerging complication. The reported prevalence varies from 8.3% to 12%.^26^–^28^ Albu et al. reported that TM patients with AHH were significantly older (median age 26 vs. 16.5 years, p: 0.007) and had higher serum ferritin levels compared to patients without AHH.^26^ TM patients rarely consult doctors due to a lack of obvious and evident symptoms of AHH. In our TM patients with AHH the presenting symptoms were a loss of libido (7 patients) or infertility (2 married patients); incomplete and/or not persistent erection (9 patients); fatigue (1 patient) and ejaculatory dysfunction (decreased or watery semen production: 11 patients). These symptoms had been present for a mean of 6 ± 2 months (range 2 – 9 months) before the first endocrine evaluation. They were previously attributed to the chronic disease itself, iron overload, low hemoglobin level, liver dysfunction, associated endocrine complications.

Early identification and management of AHH are very crucial to avoid subsequent long-term morbidity, including infertility, sexual dysfunction, osteoporosis, weakness and disturbed QOL.^1^–^4^

The concentration of serum TT reaches its maximum around 25–30 years of age and starts a slow, steady decline thereafter at a rate of about 1% per year.^8^ Furthermore, there is a 1.2% annual increase in sexual hormone-binding globulin (SHBG), which makes it unavailable to the tissues.^29^

In men, 60% of circulating testosterone is bound to sex hormone-binding globulin (SHBG), 38% is bound to albumin, and only 2% is unbound or free. Total testosterone levels might be normal with hypogonadism if the SHBG levels are increased. Levels of SHBG increase with age, causing a decrease in bioavailable testosterone.^1^–^4^ SHBG levels are elevated in patients with cirrhosis due to increased hepatic production, but the pathogenesis of this remains not fully explained. Rising levels of SHBG have been shown to correlate also with severity of fibrosis in patients with the chronic liver disease.^7^ If testosterone levels are low-normal but the clinical symptoms and signs indicate hypogonadism, measurement of serum total testosterone levels should be repeated and an SHBG level should be determined, and the bioavailable testosterone levels can be calculated.

Symptoms of androgen deficiency need to be specifically inquired about if hypogonadism is suspected, although none of these symptoms are specific to the low androgen state. Questionnaires such as Aging Male Symptom Score (AMS) and Androgen Deficiency in Aging Men (ADAM) are not recommended for the diagnosis of hypogonadism because of low specificity.^30^–^33^

Hormone replacement therapy can significantly improve the QOL of patients by restoring sexual function. The cut-off values to diagnose hypogonadism have been variable. Recently an American consensus statement reported that above 11.1 nmol/L TT is normal, below 6.9 nmol/L is diagnostic of hypogonadism, and 6.9–11.1 nmol/L is equivocal.^34^ In Europe, those figures are slightly different (12, 8 and 8–12 nmol/L respectively).^35^ The diagnosis of hypogonadism should never be based on a single testosterone level. For those patients with low testosterone, repeated measurement of testosterone level at least 1 month apart, is necessary to confirm a low testosterone level. Those with a total testosterone level between 8 – 12 nmol/l are classified as borderline. In borderline cases, the SHBG level can be measured to calculate the free testosterone using a mathematical formula (www.issam.ch), or the free testosterone can be measured using the equilibrium dialysis method. However, if further assays are not available, and patients are symptomatic, a trial of testosterone therapy can be given followed by re-assessment after three months. Those with a level above 12 nmol/l are unlikely to be hypogonadal and should not receive testosterone treatment.^8^

It is important to differentiate adult-onset HH, (characterized by frankly low serum testosterone levels in the presence of low or normal gonadotropins) from the progressive testosterone deficiency observed in a small minority of aging men, known as late-onset hypogonadism (LOH). This latter condition has been defined as a syndrome in middle-aged and elderly men reporting sexual symptoms in the presence of moderately low total testosterone levels, with variable levels of gonadotropins, which mostly involves gonadal components in its pathogenesis.^36^

The choice of therapy in males with AHH depends on the fertility requirements of the patients. When fertility is desired, gonadotropin therapy is necessary to induce spermatogenesis.^36^ Different treatment protocols can be used. The typical gonadotropin regimen combines human chorionic gonadotropin (hCG) and FSH.^37^,^38^ Two of our azoospermic TM patients received a combination of hCG and hFSH that resulted in spermatogenesis (oligoasthenozoospermia) within 6 months. Testosterone replacement is another convenient therapy if fertility is not in question.

Testosterone replacement is recommended for symptomatic classical androgen deficiency syndromes after excluding contraindications in the initial work up. Androgen deficiency can be treated using any one of the approved testosterone formulations after consideration of pharmacokinetics, patient preference, cost, and potential formulation-specific adverse effects. Adverse events are reduced high-density lipoprotein cholesterol, increased prostatic symptoms and increased cardiovascular risk.^39^ Therefore, testosterone therapy should be accompanied by a standardized monitoring plan and general health evaluation.

This study was limited by incomplete data on statural growth in thalassemia major parents. Potential causative factors of shorter final height in TM patients without AHH compared to patients with AHH include genetic factors, previous severe iron overload, desferrioxamine “toxicity”, delayed puberty and defects in the growth hormone-insulin- like growth factor-1 (GH-IGF-1) axis.

A second limitation is that our study did not localize the anatomic level of HPG axis dysfunction. Although a strong association between pituitary R2 and pituitary volume with clinical disease suggests that secondary hypogonadism is the dominant etiology, we cannot exclude tertiary hypogonadism. Further, targeted studies are needed to address these questions and to explore the potential metabolic syndrome related to hypogonadism in our TM patients.

## Conclusions

In adult eugonadal thalassaemic patients, annual screening for the development of hypogonadism should be performed. This should include history (libido, erectile function, the frequency of spontaneous erections), physical examination and biochemical assessment (SHBG and serum fasting testosterone in the early morning).

In our thalassemic patients iron overload and chronic liver disease appear to play a role in the development of AHH. Studying TM patients with AHH has become a vital and dynamic field for improving their health and QOL. Many exciting opportunities remain for further research and therapeutic development. Treatment is in the form of testosterone replacement therapy in a variety of preparations. Therapy aims to restore serum testosterone to the mid–normal range and correct symptoms and signs of androgen deficiency. However, the results and safety of long-term prospective controlled trials of testosterone therapy are still awaited.

## Figures and Tables

**Table 1:** Demographic and co-existent endocrinopathies and HCV infection data in thalassemia major patients with and without acquired hypogonadotropic hypogonadism (AHH).

Variables	Thalassemic patients without AHH (mean ± SD) (12 patients)	Thalassemic patients with AHH (mean ± SD) (11 patients)	P value

Age (yr)	34 ± 9.1	34.3 ± 8.8	0.936

Weight (Kg)	57.4 ±6.1	63 ± 7.7	0.072

Height (cm)	160.5 ± 8.7	169.5 ± 5.8	0.008

Body Mass Index (kg/m^2^)	22.3 ± 1.7	21.8 ± 1.6	0.509

HCV-ab positive (n)	9/12	9/11	0.36
HCV –RNA positive (n)	4/9	8/11	0.059

Primary hypothyroidism (n)	2	2	-

Secondary hypothyroidism (n)	2	1	-

Hypoparathyroidism (n)	2	0	-

Insulin dependent diabetes (n)	1	1	-

L1–L4 bone mass density (*Z* Score) (g/cm2)	− 2.62 ± 0.59	− 2.90 ± 0. .64	0.284

Splenectomized (n)	6/12	11/11	0.014

Poor compliance to chelation therapy (n)	3	4	0.67

**Table 2:** Relevant laboratory parameters and data used for the diagnosis of AHH and the follow up of thalassemia major patients with and without acquired hypogonadotropic hypogonadism (AHH).

Variables	Thalassemic patients without AHH (mean ± SD) (12 patients)	Thalassemic patients with AHH (mean ± SD) (11 patients)	P value

LH (IU/L)	3.9 ± 1.5	2.4 ±2.2	0.071
95% confidence interval for mean - Lower bound and upper bound	2.9 – 4.8	0.9 –3.8	-

FSH (IU/L)	3.1 ± 2.1	1.2 ± 0.9	0.008
95% confidence interval for mean - Lower bound and upper bound	1.8– 4.4	0.6–1.7	-

Total testosterone (nmol/L) (*)	18.9 ± 4	5.27 ± 3	0.001
95% confidence interval for mean - Lower bound and upper bound	16.3– 21.5	3.1– 7.4	-

IGF1 (ng/ml)	87.6 ± 57.1	53.3 ± 18.7	0.071
95% confidence interval for mean - Lower bound and upper bound	51.3–123.9	40–66.7	-

Global Heart T2* (msec)	36.5 ± 12.5	17.5 ± 6.9	0.004
(8 vs.6). 95% confidence interval for mean - Lower bound and upper bound	26.1 – 46.9	10.3 – 24.7	-

Left ventricular ejection fraction (%) (11 vs. 8)	62.4 ± 3.9	61.5± 5.3	0.676

Liver iron concentration (mg Fe/g dry wt) (9 vs. 8)	8.7 ± 5.9	9.7 ± 10.3	0.817
Liver iron concentration > 7 mg/g dry weight (n)	5/9	3/8	0.66

Albumin (g/L)	61.1± 6.8	58 ± 5.6	0.248

ALT (U/L)	43.3 ± 20.5	85 ± 66.2	0.069

ϒ-GT (U/L)	26.2 ± 15.2	77.7 ± 82.9	0.016

Alkaline phosphatase (IU/L)	218 ± 71.7	277 ± 120.9	0.260

International normalised ratio [INR] (12 vs.9)	1.2 ± 0.12	1.2 ± 0.14	0.937

Prothrombin time (seconds)	8 ± 0.6	7.9 ± 0.4	0.487

Serum ferritin (ng/ml)	968.4 ± 775.4	1697.6 ± 1472.5	0.525
95% confidence interval for mean - Lower bound and upper bound	475.7–1461.1	708.3 – 2686.8	-
Serum ferritin level > 2000 ng/ml (n)	1/12	5/11	-

*AHH - Adult-Onset Hypogonadotropic Hypogonadism; Normal values* : LH: 2.6–6.7 IU/L; FSH: 1.3 –7.4 IU/L; Total testosterone (2.5–97.5th percentile): 8.7–31.7 nmol/L (*) To convert nmol/L to ng/mL multiply by 0.29; IGF-1: 95.6–366.7 ng/ml for ages 25 to 39 yrs, 60.8–297.7 ng/ml for 40 to 59 yrs; Albumin: 35–55 g/L; Alanine Aminotransferase (ALT): 5–40 U/L; γ Glutamyl transferase: 10–49 U/L; Alkaline phosphatase: 25–90 IU/L; INR: 0.9–1.2; Prothrombin time: 10–13 s; Serum ferritin: 20–200 ng/ml.
